# Acute Right Coronary artery Occlusion During Cooled-Tip Radiofrequency Ablation of the Cavotricuspid Isthmus

**DOI:** 10.1016/s0972-6292(16)30607-6

**Published:** 2013-03-07

**Authors:** Eduardo Castellanos, Jesus Almendral

**Affiliations:** Unidad de Electrofisiologia Cardiaca y Arritmologia Clinica. Grupo Hospital de Madrid. Universidad CEU-San Pablo. Madrid, Spain

**Keywords:** Right Coronary artery Occlusion, Cooled-Tip Radiofrequency Ablation, Cavotricuspid Isthmus

## Abstract

Cooled-tip radiofrequencycatheter ablation of the cavotricuspid isthmus, performed after pulmonary vein isolation, resulted in acute occlusion of the posterolateral branch of the right coronary artery in a 49-year-old male with previously known right coronary artery disease. The occlusion was successfully stented. It is conceivable that previously diseased coronary arteries are more prone to be damaged during ablation.

## Introduction

The incidence of coronary injury secondary to radiofrequency catheter ablation (RFA) procedures in patients with atrial arrhythmias is very low [1]. The cavotricuspid isthmus (CTI) is the target of typical atrial flutter (AFL) ablation. Despite the proximity of the right coronary artery (RCA) to the CTI, only few reports of direct damage to the RCA resulting in myocardial infarction (MI) during CTI catheter ablation have been published [1-5]. There is controversy whether to use conventional or irrigated tip catheters for CTI ablation. However, since most atrial fibrillation ablation procedures are performed with irrigated tip catheters it is tempting to use this type of catheters when CTI ablation is performed as part of an atrial fibrillation ablation procedure. We report a case in which acute occlusion of the RCA occurred in this context.

## Case Report

A 49-year-old man, truck driver, with a history of hypertension, non-insulin-dependent diabetes, obstructive sleep apnea and coronary disease (stent implanted at the RCA two years before), was referred to our institution for catheter ablation. He complained of recurrent episodes of palpitations and dizziness during the previous six months, unresponsive to atenolol (50 mg/d). He presented one episode of syncope preceded by palpitations on atenolol. The ECG showed sinus bradycardia, and the Holter ECG documented multiple symptomatic episodes of paroxysmal atrial fibrillation (AF) and typical AFL. Transthoracic echocardiography and treadmill test were normal.

**Figure 1:**
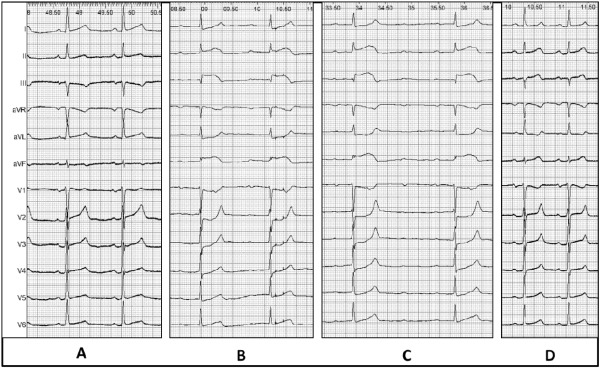
Twelve-lead ECGs: A, at the beginning of the procedure B, at the end of the ablation procedure: note the PR prolongation and the ST-segment elevation, particularly prominent in lead III andaVF. C, 2 minutes after ablation, showing advanced AV block, and more prominent ST-segment elevation. D, after coronary revascularization with normalization of the ST elevation.

A decision was made to proceed with catheter ablation of AF as well as CTI ablation. The patient underwent electrical isolation of the 4 pulmonary veins under general anesthesia, using three-dimensional electroanatomic imaging (NavX, St. Jude Medical, St Paul, Minnesota USA). We used a 3.5 mm-tip external irrigated ablation catheter (EZ Steer™ ThermoCool® Catheter, Biosense Webster, Diamond Bar, CA), and a powered controlled setting (Atakr® II Generator, Medtronic), with a maximum power of 35 W (30W for the posterior wall of the left atrium) and a temperature limit of 43ºC. The procedure was successful and isolation of the four pulmonary veins was obtained without complications. The same catheter was used later for the CTI ablation with the same parameters. RFA of the CTI was performed in a point by point fashion during sinus rhythm. After 7 applications, bidirectional isthmus block was achieved. Immediately after documentation of CTI block, we noted sudden PR prolongation with ST-segment elevation in the inferior leads followed by progressive second degree AV block (Figure 1, panels A-C).

**Figure 2:**
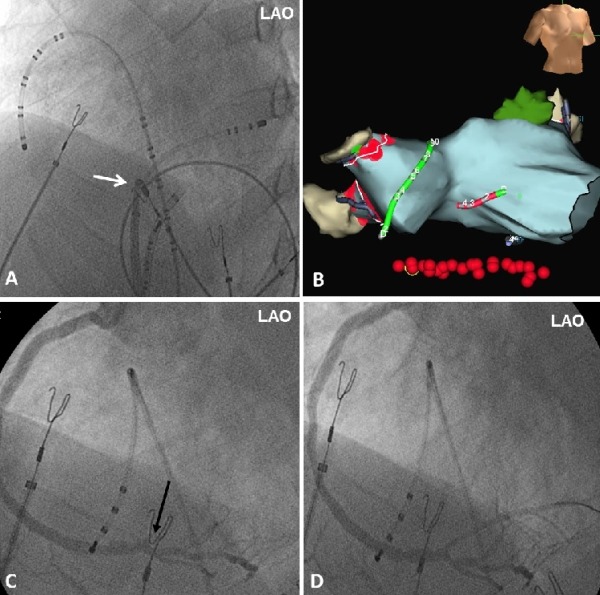
Panel A and B show the left anterior oblique (LAO) fluoroscopic view and the three-dimensional electroanatomic imaging respectively with the position of the catheters at the end of the ablation procedure. The red dots represent the radiofrequency applications around the pulmonary veins, and through the cavotricuspid isthmus (three-dimensional red dots). A quadripolar catheter is placed in the coronary sinus, a multipolar catheter along the lateral right atrium and the ablation catheter (white arrow) in the cavotricuspid isthmus. Panel C shows the occlusion of the posterolateral branch of the RCA (black arrow). Panel D shows the end angiographic results post stenting.

A tetrapolar electrode was positioned at the apex of the right ventricle for temporary pacing, and the other catheters were removed. A coronary angiogram was performed, showing no restenosis at the stent previously implanted at the proximal RCA and a total occlusion of the origin of the posterolateral branch of the RCA by a recent thrombus (Figure 2). The RCA was extensively calcified. A guidewire was introduced and the vessel was dilated; a drug-eluting stent was implanted with restoration of distal blood flow of the posterolateral branch. The patient recovered a normal AV conduction with normalization of the ST segment (Figure 1, panel D). Troponin C peaked at 1.61 ng/mL the following day. The echocardiogram before discharge showed a mild inferior wall hypokinesis and normal left ventricular ejection fraction.

Enoxaparin, aspirin and clopidogrel, were given after the procedure. Enoxaparin was later substituted by acenocumarol. The clinical course was uneventful. Six months later, the patient was asymptomatic without further episodes of recurrent AF or AFL.

## Discussion

We present the case of RCA occlusion immediately after CTI ablation performed as part of an atrial fibrillation ablation procedure. The occurrence at the site of and immediately after CTI ablation strongly suggests a relationship between CTI ablation and RCA occlusion, although coronary artery embolization of a left atrial thrombus cannot be completely excluded.

Catheter ablation is mostly performed at the present time with large-tip or irrigated tip ablation electrodes in order to produce larger and deeper lesions and increase efficacy. Although preliminary studies did not describe a significant increase in complications using larger and irrigated tip catheters, several cases in the literature reported RCA injury related with CTI ablation with these catheters [1-5]. Some anatomic and pathophysiological circumstances can facilitate coronary artery injury:

***1) Anatomical factors:*** The proximity between the target for ablation and the coronary vessels; some reported cases emphasized that the risk of coronary injury is higher with a septal or lateral CTI approach. The RCA is located close to the CTI and coronary sinus ostium, and the distance between the large posterolateral branch of the RCA and the ostium of the coronary sinus-middle cardiac vein has been reported to be <2 mm in 20% of the patients. So damage to the RCA could be more frequent when the catheter falls into a large coronary sinus [4], or near the middle cardiac vein [1] during the ablation (detected by an impedance rise). Other retrospective studies in patients who had cardiac computed tomography for different indications, revealed that the proximity between the RCA and the CTI is maximal at the atrial aspect of the lateral isthmus, compared to more septal or ventricular sites.

***2) Coronary circulation:*** A lack of sufficient cooling effect in patients with coronary artery disease with a severe upstream coronary stenosis has also been suggested as a situation that could increase the likelihood of coronary injury during RFA. Furthermore, even in patients without coronary artery disease a depression of the coronary flow reserve has been observed associated with impairment in myocardial perfusion during CTI ablation. Our patient had coronary artery disease with a previous stent in the proximal RCA. Although the stent did not show stenosis, the whole vessel was diseased and calcified, which could have made it more prone to damage.

The clinical presentation is also of note. Different clinical presentations have been reported, with or without chest pain, inferior ST elevation [2] and PR prolongation [5]. Ventricular fibrillation during the procedure may be the first clinical presentation [4]. In one reported case, the patient died of another cause and an intramural hemorraghe of the RCA was found at autopsy [3]. Since our patient was anesthetized, pain was not reported and the clues to the diagnosis were abnormalities in AV nodal conduction and ST segment elevation.

## Conclusion

Although CTI catheter ablation is a low-risk procedure, injury to the RCA can exceptionally occur. Since certain anatomical and clinical factors could potentially be associated with an increased risk, they should be taken into consideration.

## References

[R1] Roberts-Thomson KC (2009). Coronary artery injury due to catheter ablation in adults. Circulation.

[R2] Ouali S (2002). Acute coronary occlusion during radiofrequency catheter ablation of typical atrial flutter. J Cardiovasc Electrophysiol.

[R3] Weiss C (2002). Can radiofrequency current isthmus ablation damage the right coronary artery? Histopathological findings following the use of a long (8 mm) tip electrode. Pacing Clin Electrophysiol.

[R4] Mykytsey A (2010). Right coronary artery occlusion during RF ablation of typical atrial flutter. J Cardiovasc Electrophysiol.

[R5] Caldwell JC (2010). Right coronary artery damage during cavotricuspid isthmus ablation. Pacing Clin Electrophysiol.

